# Single Seed Microbiota: Assembly and Transmission from Parent Plant to Seedling

**DOI:** 10.1128/mbio.01648-22

**Published:** 2022-10-12

**Authors:** Guillaume Chesneau, Béatrice Laroche, Anne Préveaux, Coralie Marais, Martial Briand, Brice Marolleau, Marie Simonin, Matthieu Barret

**Affiliations:** a Univ Angers, Institut Agro, INRAE, IRHS, SFR QUASAV, Angers, France; b Université Paris-Saclay, INRAE, MaIAGE, Jouy-en-Josas, France; University of Toronto

**Keywords:** seed, microbiota, community assembly, seedling transmission, selection, neutral models

## Abstract

The seed acts as the primary inoculum source for the plant microbiota. Understanding the processes involved in its assembly and dynamics during germination and seedling emergence has the potential to allow for the improvement of crop establishment. Changes in the bacterial community structure were tracked in 1,000 individual seeds that were collected throughout seed developments of beans and radishes. Seeds were associated with a dominant bacterial taxon that represented more than 75% of all reads. The identity of this taxon was highly variable between the plants and within the seeds of the same plant. We identified selection as the main ecological process governing the succession of dominant taxa during seed filling and maturation. In a second step, we evaluated the seedling transmission of seed-borne taxa in 160 individual plants. While the initial bacterial abundance on seeds was not a good predictor of seedling transmission, the identities of the seed-borne taxa modified the phenotypes of seedlings. Overall, this work revealed that individual seeds are colonized by a few bacterial taxa of highly variable identity, which appears to be important for the early stages of plant development.

## INTRODUCTION

The seed microbiota allows for the transmission of microbial communities between plant generations (1) and is a key factor influencing both seed vigor and seedling development, two essential steps for crop establishment. Several studies focused on the role of the mature seed microbiota in releasing dormancy (2), improving germination (3), or protecting against damping-off (4). In addition, seed microbiota may have longer-term consequences on crop establishment by promoting the establishment of soilborne plant-beneficial microorganisms (5) or by limiting the incidence of soilborne plant pathogens (6). However, all of the above-mentioned studies characterized the microbiota of dried mature seeds, while limited knowledge is available on the dynamics of microbiota assembly during seed development (7). A consequence of this knowledge gap is that the origin, timing of arrival, and succession of seed-associated microbial taxa is mostly unknown, as are their future consequences on seed vigor and seedling development.

To date, the characterization of seed microbiota structure has been performed on about 50 plant species (8), including major crops such as barley (9), bean (10), rapeseed (11, 12), rice (13), tomato (14), and wheat (15). Selection by the local environment or by the host significantly modulates seed microbiota composition (10, 12, 16). Other ecological processes, such as dispersal by pollinators (17) and ecological drift (18) are also important drivers of seed community assembly (19). However, in most studies, seed microbiota was characterized on large seed samples from 10 to 1,000 seeds. One reason for using seed lots rather than individual seeds is related to the technical difficulties of collecting enough microbial DNA from a single seed, as these are usually colonized by a small bacterial population size (20). Another rationale for using seed samples is related to historical studies carried out in seed phytopathology. Indeed, seed-transmitted plant pathogens are generally distributed within a Poisson distribution (21), and increasing sampling size increases the probability of detection. While working on seed lots is valid for improving plant pathogen detection, this sampling procedure artificially increases seed microbiota richness and, more importantly, does not allow for the estimation of seed-to-seed variability.

The few studies carried out at the individual seed level or small seed samples (i.e., less than 10 seeds) through culture-dependent (20, 22, 23) or culture-independent (24–26) approaches have revealed a low microbial richness. It has even been proposed that each seed was either not colonized or was colonized by only one endophytic microorganism (22). The authors of the latter study propose a hypothesis, the “primary symbiont hypothesis”, which states that endophyte microbial transmission in seeds is bottlenecked as a result of plant defenses and interactions among seed-transmitted microorganisms (22). However, this hypothesis has still not been confirmed at the whole individual seed microbiota level, which includes endophytes and epiphytes. In addition, the order and routes of arrival of these “primary symbionts” on seeds, as well as the ecological processes involved in their assembly, remain unexplored.

The interest in studying and characterizing the seed microbiota is to improve the phenotype of the future plant. To this end, it is essential to know the future of seed-associated taxa on the seedlings as well as the impact they may have on the plant phenotype, whether beneficial or harmful. However, the presence of a microorganism on or in seeds is not a guarantee of transmission to the seedling (27). To date, studies focusing on the dynamics of microbial communities during germination and emergence have been performed on several subsamples of the same seed lot (9, 15, 28–30). However, in order to truly assess the transmission of seed-borne bacterial taxa to seedlings, it is necessary to work at the individual seed-seedling level in a nondestructive way.

In this context, this study aimed to characterize the bacterial diversity of individual seeds during their development. Several fundamental questions were addressed: (i) whether the first microorganisms that colonize the seed during its development are the ones that will be established on the mature seeds; (ii) what the main seed transmission pathways employed by these seed-borne taxa are; and (iii) what the ecological processes involved in seed microbiota assembly are. In the second part of this study, we evaluated the dynamics of seed-borne taxa during individual seedling emergence. Specifically, we focused on the following two questions: (iv) whether the initial abundance of seed-borne taxa was a good predictor of their transmission to seedlings and (v) what their impacts on seedling phenotypes are.

To answer these questions, we worked with the seeds of common bean (Phaseolus vulgaris) and radish (Raphanus sativus), two plant species whose seed microbiota have been extensively characterized (7, 10, 18). We estimated the microbiota composition of single seeds of different individual plants during seed filling and maturation. We also inferred the origins of seed-associated taxa via the sampling and sequencing of flowers, vascular tissues, and atmosphere microbiota. In a second step, we evaluated the transmission of seed-borne bacteria by seedlings via sampling individual seed and seedling microbiota in a nondestructive way. Finally, we assessed the impact of the seed microbiota on seedling emergence and confirmed the impacts of some selected taxa on seedling fitness. The results of this study provide essential information on the seed microbiota assembly, and these results can be used in the development of microbial-based solutions for the improvement of seed vigor and protection against pathogens.

## RESULTS

### Experiment 1: Culture-based enrichment of seed-associated bacterial taxa.

The sequencing of samples with low microbial biomass, such as seeds, is subject to a low signal to noise ratio as a result of weak amounts of DNA starting material (31). To capture the bacterial diversity at a single seed level, an experimental strategy based on culture-based enrichment was employed.

**FIG 1:**
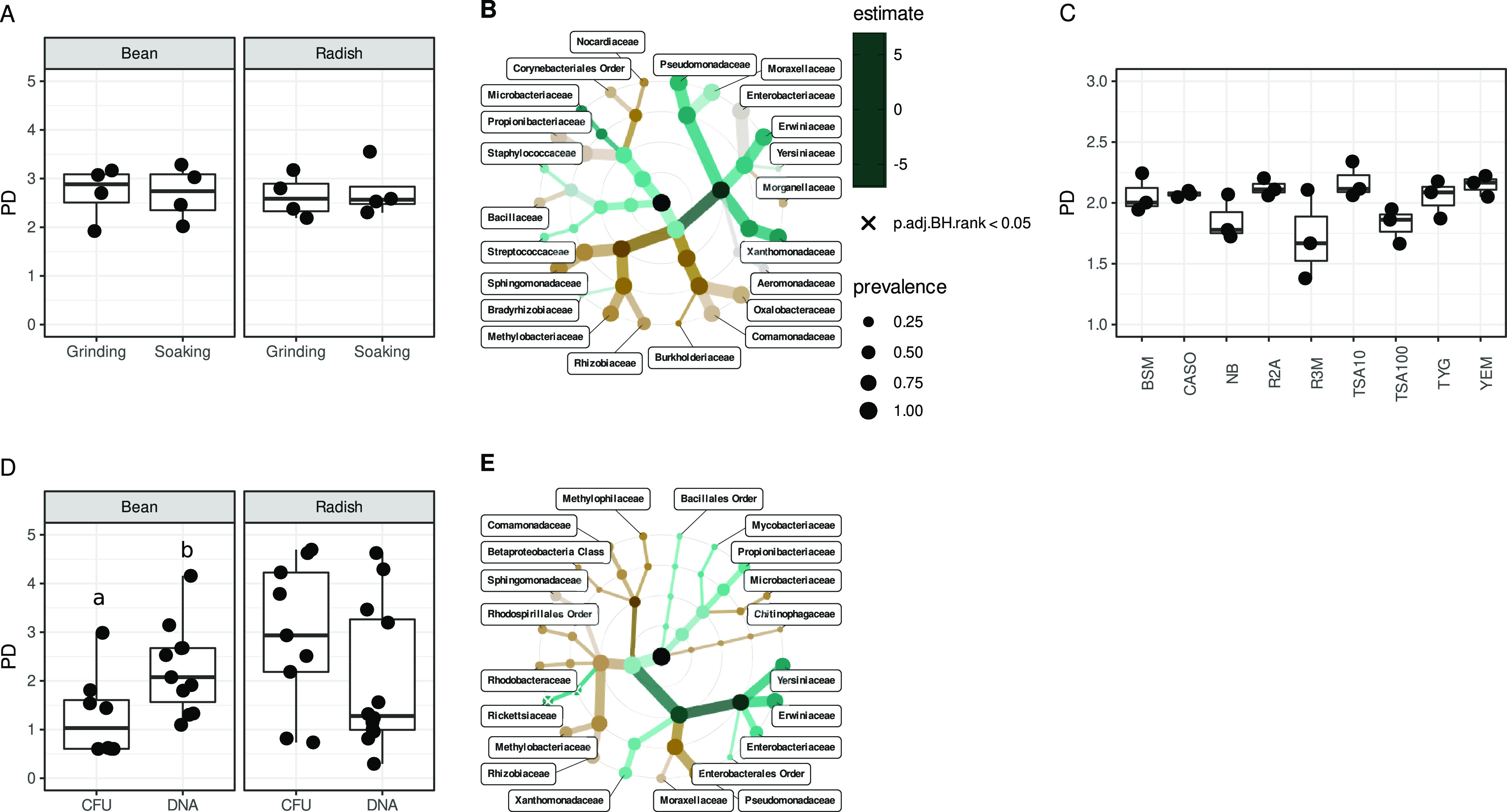
Culture-based enrichment of seed-associated bacterial taxa. (A) Phylogenetic diversity estimation of seed bacterial communities following grinding or soaking. (B) Changes in the relative abundance of bacterial families following grinding or soaking. (C) Phylogenetic diversity estimation of bacterial colony forming units (CFU) collected on nine different media. (D) Phylogenetic diversity estimation of bacterial taxa recovered with (CFU) and without (DNA) enrichment on TSA10 media. Letters indicate statistically significant changes (*P* < 0.05) in phylogenetic diversity. (E) Changes in the relative abundance of bacterial taxa recovered with (CFU) and without (DNA) enrichment on TSA10 media.

Two methods (i.e., seed soaking or seed grinding) for recovering bacterial cells from bean and radish seed samples were first compared. The amplicon sequencing of DNA samples obtained with either of these methods revealed no significant differences in bacterial phylogenetic diversity (Fig. 1A). The abundance of bacterial families was not significantly modulated by the method of bacterial cell recovery (Fig. 1B). Therefore, we selected seed soaking, as this method released fewer antimicrobial compounds in the suspension in comparison to seed grinding.

Then, seed soaking suspensions were spread on nine culture media used to isolate plant-associated bacteria. Tryptic soy agar 1/10 strength (TSA10) allowed the greatest diversity of seed bacterial isolates to be obtained (Fig. 1C) and was thus chosen.

Finally, the bacterial community profiles obtained before and after culture-enrichment on TSA10 were examined. The bacterial phylogenetic diversity estimated before or after enrichment on TSA10 was not significantly different for the radish seed samples (Fig. 1D). In contrast, a significant decrease (*P* < 0.05) in phylogenetic diversity was observed for the bean seed samples after enrichment on TSA10 (Fig. 1D). Amplicon sequence variants (ASVs) affiliated with Rickettsiaceae were significantly (*P* < 0.05) underestimated following culture-enrichment (Fig. 1E). However, the other bacterial families were not significantly impacted by the enrichment process (Fig. 1E).

Overall, these results highlighted that the vast majority of seed-borne bacterial taxa were efficiently captured by the culture-based enrichment performed on TSA10. Therefore, for the rest of the study, we systematically performed culture enrichment of our single seed samples on TSA10 before DNA extraction and sequencing.

### Experiment 2: Assembly and structure of the microbiota during individual seed development.

**FIG 2:**
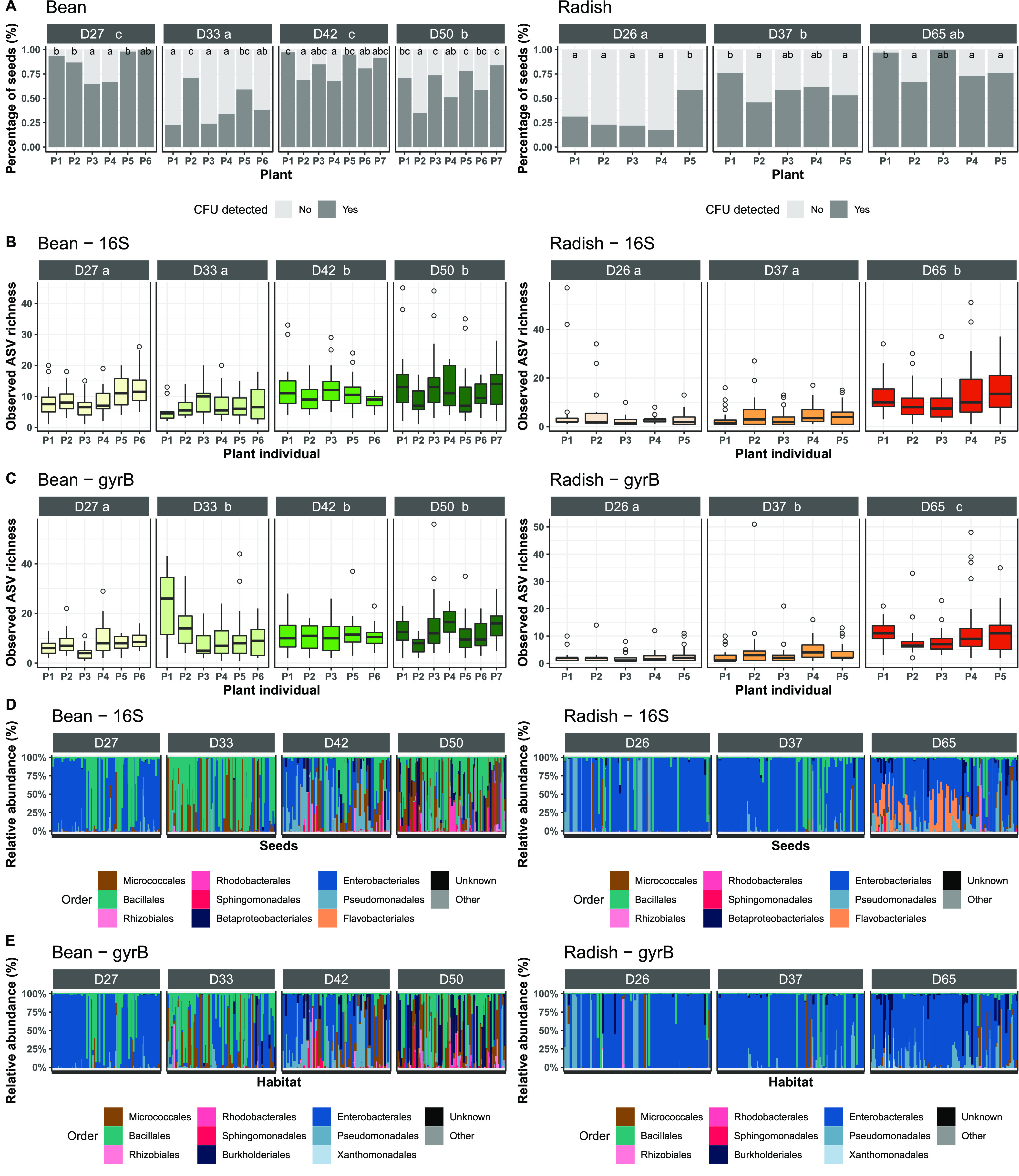
Structure of the bacterial fraction of the seed microbiota. (A) Percentage of seeds with (dark gray) or without (light gray) CFU detected. (B and C) Number of amplicon sequence variants (ASVs) per seed (richness), estimated with 16S rRNA genes (B) or *gyrB* (C). Seeds were grouped according to their plant of origin (*x* axis). (D and E) Relative abundance of bacterial orders for each individual seed, according to 16S rRNA genes (D) or *gyrB* (E). White dotted lines represented individual plants. The names in each frame correspond to the stages sampled during seed development. The letters (a, b, c) indicate the significance level (*P* < 0.01).

**(i) Individual seeds are associated with low bacterial richness.** Seeds from bean and radish were collected aseptically during seed filling and maturation, two stages defined by measuring seed water content (Fig. S1A) (32). On average, bacterial colony forming units (CFU) were detected in 43% to 84% of bean seeds per stage and in 30% to 82% of radish seeds (Fig. 2A). Variation in the percentage of seeds containing bacteria was associated with the sampling stage as well as between individual plants for both plant species (*P* < 0.01) (Fig. 2A).

10.1128/mbio.01648-22.2 FIG S1 Experimental design used to monitor the assembly of the bacterial communities during seed development. (A) Seed water content (%) associated with samples collected during bean (left) and radish (right) seed development. The letters denote statistical significance, as evaluated via the Kruskal-Wallis test followed by Dunn’s *post hoc* test (*P* < 0.01). (B) Number of samples processed at each stage. (C) Sampling of flowers, stems, and atmosphere, which could represent the main inoculum sources for the seed taxa. Download FIG S1, TIF file, 0.3 MB. Copyright © 2022 Chesneau et al. 2022 Chesneau et al. https://creativecommons.org/licenses/by/4.0/ This content is distributed under the terms of the Creative Commons Attribution 4.0 International license.

Few bacterial taxa were detected per individual seed. Median richness values of 8 and 4 16S rRNA gene ASVs were observed on bean and radish seeds, respectively (Fig. 2B). With regard to the data obtained with *gyrB*, medians of 9 and 3 ASVs were observed in bean and radish seeds (Fig. 1C). Bacterial richness significantly (*P* < 0.01) increased during the seed development of both plant species, regardless of the molecular markers employed (Fig. 1B and C). Within each sampling stage, a high variability in richness was observed between seeds (Fig. 1B and C). The variation in richness was accompanied by a high variation in taxonomic composition (Fig. 1D and E). The changes in phylogenetic composition between the seed bacterial communities were significantly (*P* < 0.001) explained by the seed development stages for bean (16S rRNA genes: 18.3%; *gyrB*: 16.1%,) and radish (16S rRNA genes: 15.3%; *gyrB*: 7.4%). In addition, the variation in phylogenetic composition was also significantly (*P* < 0.001) associated with plant interindividual variations in bean (16S rRNA genes: 26.5%; *gyrB*: 26.1%) and radish (16S rRNA genes: 28.5%; *gyrB*: 20.0%).

**(ii) The origin of seed-associated taxa.** Transmission routes of seed-associated bacterial taxa were estimated through the additional sampling of the atmosphere, flowers, and stems (Fig. S1C). The percentage of seed taxa (i.e., *gyrB* ASVs) undetected in the atmosphere, flowers, and stems was higher than 80% for both plant species at each sampling time (Fig. S3). The remaining seed taxa were mostly detected in the atmosphere, compared to the other habitats sampled (Fig. S3).

10.1128/mbio.01648-22.4 FIG S3 Origin of seed-borne taxa. (A and B) Number of *gyrB* ASVs associated with bean (A) and radish (B) seeds that were detected in other sampled habitats: flowers, stems, and atmosphere. Unknown (grey) corresponds to seed-associated taxa not detected in any source habitat sampled. The right panel is a zoom of the left panel. Download FIG S3, TIF file, 0.2 MB. Copyright © 2022 Chesneau et al. 2022 Chesneau et al. https://creativecommons.org/licenses/by/4.0/ This content is distributed under the terms of the Creative Commons Attribution 4.0 International license.

**FIG 3:**
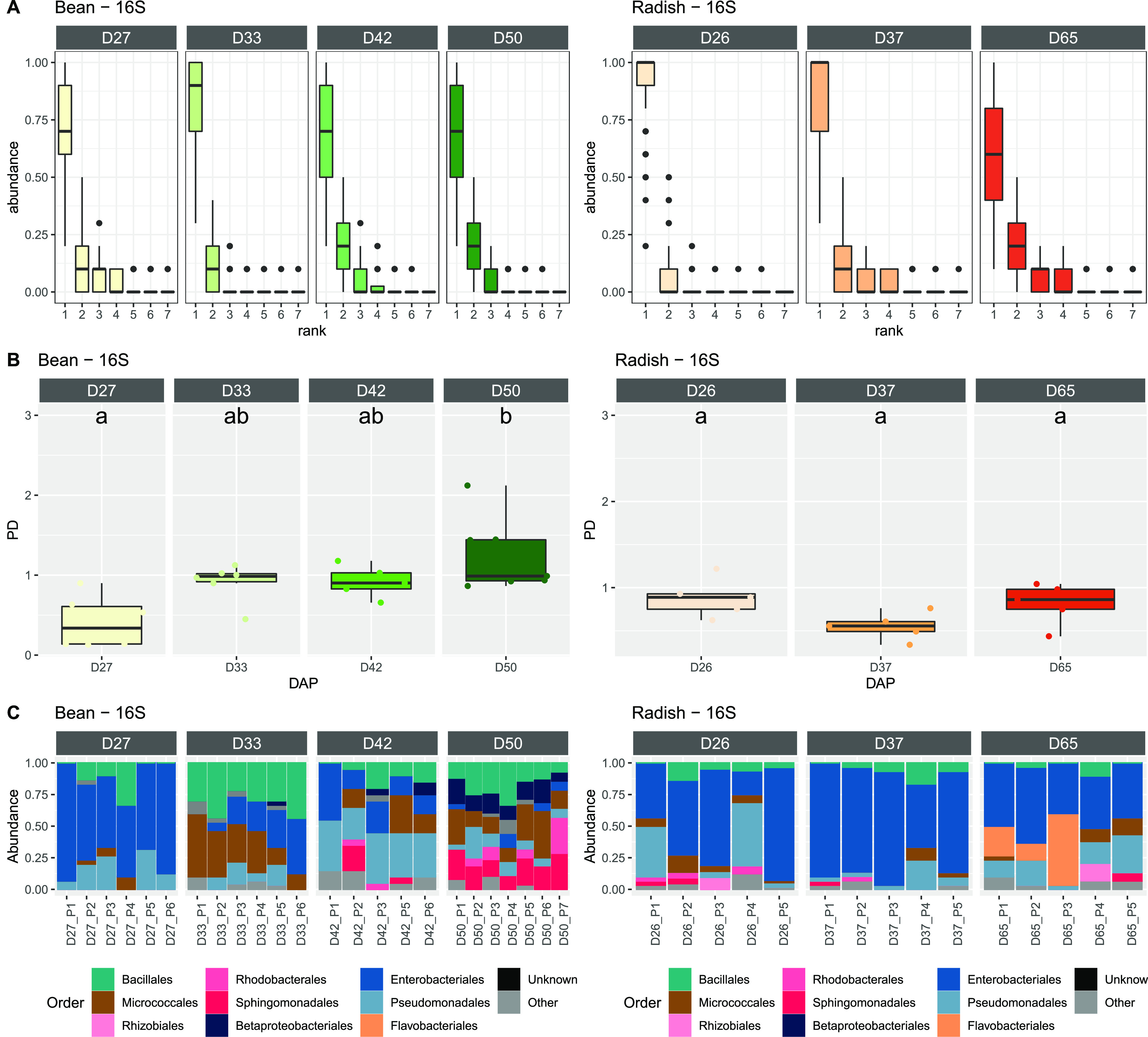
Dominant bacterial taxa within the seed microbiota, based on 16S rRNA genes. (A) Rank-abundance curves of bacterial ASVs associated with individual seeds. (B) Faith’s phylogenetic diversity of rank 1 ASV agglomerated at the plant level. (C) Relative abundance of bacterial orders calculated from rank 1 ASV agglomerated at the plant level. The names in each frame correspond to the stages sampled during seed development. The letters (a, b, c) indicate the significance level (*P* < 0.01).

**(iii) A seed is associated with one dominant bacterial taxon.** Rank-abundance curves indicated the dominance of one taxon per bean and radish seed at each developmental stage (Fig. 3A; Fig. S2A). These dominant taxa had mean relative abundance valuse of 77.1% (+ 22.0%) with the 16S rRNA genes (Fig. 3A) and 75.4% (+ 22.8%) with *gyrB* (Fig. S2A). The dominant taxa were distributed across more than 15 bacterial orders and represented 94 16S rRNA gene ASVs (Fig. S4) and 286 *gyrB* ASVs (Fig. S5). This high taxonomic variability indicates that the culture-based enrichment strategy does not systematically select certain bacterial taxonomic groups.

10.1128/mbio.01648-22.3 FIG S2 Dominant bacterial taxa within the seed microbiota based on *gyrB*. (A) Rank-abundance curves of bacterial ASVs associated with individual seeds. (B) Faith’s phylogenetic diversity of rank 1 ASV agglomerated at the plant level. (C) Relative abundance of bacterial orders calculated from rank 1 ASV agglomerated at the plant level. The names in each frame correspond to the stages sampled during seed development. The letters (a, b, c) indicate the significance level (*P* < 0.01). Download FIG S2, TIF file, 0.5 MB. Copyright © 2022 Chesneau et al. 2022 Chesneau et al. https://creativecommons.org/licenses/by/4.0/ This content is distributed under the terms of the Creative Commons Attribution 4.0 International license.

10.1128/mbio.01648-22.5 FIG S4 Phylogenetic diversity of seed-borne dominant taxa, based on 16S. Neighbor-joining phylogenetic tree of dominant 16S ASV. The occurrence of seed-borne ASVs within a specific plant is represented by a circle whose size is proportional to its relative frequency. Tips labels represent taxonomy at the order level. Download FIG S4, TIF file, 0.3 MB. Copyright © 2022 Chesneau et al. 2022 Chesneau et al. https://creativecommons.org/licenses/by/4.0/ This content is distributed under the terms of the Creative Commons Attribution 4.0 International license.

10.1128/mbio.01648-22.6 FIG S5 Phylogenetic diversity of seed-borne dominant taxa, based on *gyrB*. Neighbor-joining phylogenetic tree of dominant *gyrB* ASV. The occurrence of seed-borne ASVs within a specific plant is represented by a circle whose size is proportional to its relative frequency. Tips labels represent taxonomy at the order level. Download FIG S5, TIF file, 1.3 MB. Copyright © 2022 Chesneau et al. 2022 Chesneau et al. https://creativecommons.org/licenses/by/4.0/ This content is distributed under the terms of the Creative Commons Attribution 4.0 International license.

Phylogenetic diversity and taxonomic composition indicated the succession of dominant taxa over seed filling and the maturation of bean seeds. Indeed, the phylogenetic diversity of the dominant taxa per plant significantly (*P* < 0.05) increased during bean seed development (Fig. 3B). Moreover, a clear shift in bacterial taxonomic composition was observed during bean seed development, with a decrease in the occurrence of dominant taxa being observed to be affiliated with Enterobacterales (Fig. 3C; Fig. S2C). For radish, no changes in phylogenetic diversity or taxonomic composition were observed during the sampled stages (Fig. 3C; Fig. S2C).

**(iv) Dispersion is less important than local seed processes.** Since the dominant seed-associated taxa were specifically associated with one plant individual (Fig. S4, S5), the proportions of observed ASVs within each seed were aggregated at the plant level to form a species-proportion distribution. We compared the resulting distribution with the observed species proportions predicted by an adapted neutral model (see Materials and Methods).

**FIG 4:**
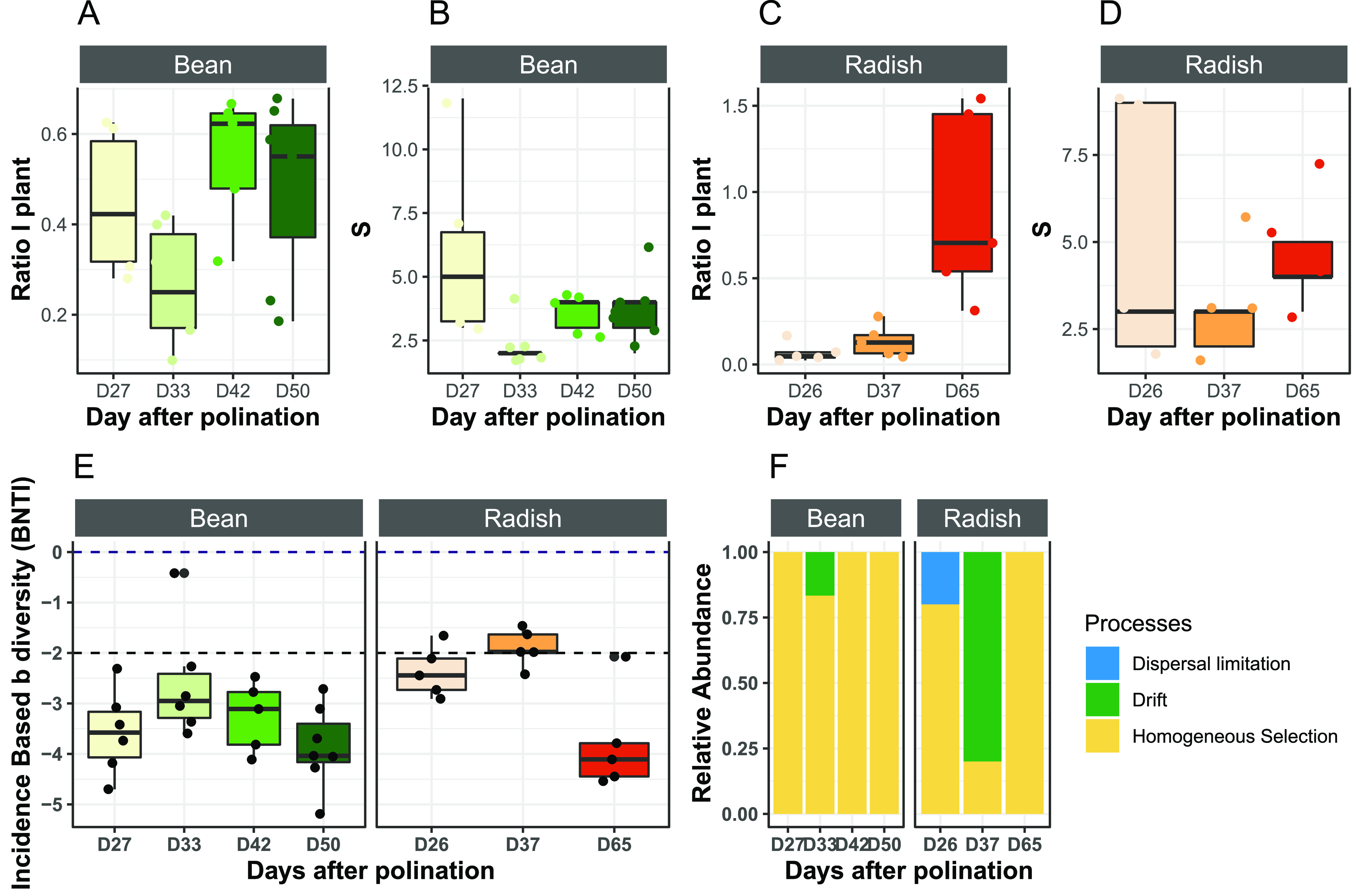
Ecological processes involved in seed microbiota assembly. (A–C). The ratio I is used to estimate the relative importance of dispersal over seed internal processes. (B–D). Distribution of S (richness) as a function of the sampling day. (E) The β-nearest taxon index (βNTI), calculated to assess the deviation from the null expectation. (F) Relative importance of species-sorting (homogeneous selection), dispersal limitation, or ecological drift in the assembly of seed bacterial communities at the plant level. Analyses were performed with 16S rRNA genes (D). Panels A–D were calculated at the individual seed level, while panels E and F were calculated at the plant level.

Species-proportion distributions are considered first order approximations for the assessment of the relative importance of dispersal over local processes (i.e., ecological drift or selection) through the ratio I. According to this ratio, dispersion was less important than local processes in bean seeds (Fig. 4A; Fig. S6A) and radish seeds (Fig. 4C; Fig. S6C). A comparison of this ratio between the stages of bean seed development indicated no change in the relative importance of dispersion and local seed processes over time (Fig. 4A; Fig. S6A). In contrast with the radish seeds, I increased significantly (*P* = 0.012 for the 16S rRNA genes and *P* = 0.05 for *gyrB*) between D37 and D65 (Fig. 4A; Fig. S6C).

10.1128/mbio.01648-22.7 FIG S6 Ecological processes involved in seed microbiota assembly. (A–C) The ratio I is used to estimate the relative importance of dispersal over seed internal processes (see Materials and Methods). (B–D) Distribution of S (richness) as a function of the sampling day. (E) β-nearest taxon index (βNTI), calculated to assess the deviation from the null expectation. (F) Relative importance of species-sorting (homogeneous selection), dispersal limitation, or ecological drift in the assembly of seed bacterial communities at the plant level. The analyses were performed with *gyrB* (D). Panels A–D were calculated at the individual seed level, while panels E and F were calculated at the plant level. Download FIG S6, TIF file, 0.3 MB. Copyright © 2022 Chesneau et al. 2022 Chesneau et al. https://creativecommons.org/licenses/by/4.0/ This content is distributed under the terms of the Creative Commons Attribution 4.0 International license.

**(v) Selection drives the local seed microbiota assembly at the plant level.** According to the species-proportion distributions, local processes (ecological drift or selection) were more important than dispersal for the assembly of the bacterial communities associated with seeds. To estimate the relative importance of selection and ecological drift in the local assembly of the seed microbiota null model, more specific, abundance-based β-null deviation measures were calculated (33). With respect to the data obtained from the bean seeds with 16S rRNA genes and *gyrB*, the β-nearest taxon index (βNTI) values negatively deviated from the null expectation (Fig. 4E; Fig. S6E). βNTI values less than −2 are considered significantly different from the null expectation (33). All of the βNTI values were below this threshold, indicating that the phylogenetic composition of the bean seed communities aggregated at the plant level are more similar than would be expected by chance and that this similarity could be driven by selection (34).

Regarding the bacterial communities associated with radish seeds, the βNTI values were distributed around −2 with the 16S rRNA genes and *gyrB* for the two first sampling stages and then less than −2 at the final mature seed stage (Fig. 4E; Fig. S6E). Therefore, it seems that selection had less of an influence on the assembly of radish seed communities, at least on the early seed developmental stages, in comparison to the bean seed communities. By the quantitative process estimates (QPE) framework (33), the relative importance of other ecological processes were estimated during seed microbiota assembly. Based on this framework, it seems that ecological drift occurred during the early assembly stages (D26 and D37) of the radish seed microbiota (Fig. 4F; Fig. S6F). At the late seed maturation stage of radish (D65), selection became the principal process of community assembly (Fig. 4F; Fig. S6F).

### Experiment 3: Seedling transmission of seed-borne taxa and their influences on seed vigor.

We next characterized which seed-borne bacterial taxa were transmitted to the seedling. The dynamics of the seed microbiota during emergence were analyzed in soil-less conditions using a nondestructive sampling procedure, which made it possible to monitor these dynamics at the level of the individual seedling. We analyzed only the samples where CFUs were recovered for seeds and seedlings; therefore, nongerminating seeds or seeds without detectable CFUs were excluded (Fig. S7A).

10.1128/mbio.01648-22.8 FIG S7 Comparison of seed and seedling microbiota profiles. (A) Number of nongerminated seeds (dark grey), abnormal seedlings (grey), and normal seedlings (light grey). (B) Average bacterial population size (log_10_ CFU) associated with the seeds and seedlings. (C) Average richness (estimated with 16S rRNA gene ASVs) in the seeds and seedlings. (D) Taxonomic profile (order level, 16S rRNA genes) of the seed and seedling microbiota. The *P*-values were derived from a Wilcoxon rank sum test. Download FIG S7, TIF file, 0.5 MB. Copyright © 2022 Chesneau et al. 2022 Chesneau et al. https://creativecommons.org/licenses/by/4.0/ This content is distributed under the terms of the Creative Commons Attribution 4.0 International license.

During the transition from seed to seedling, the median bacterial population size increased by 1.4 to 6.0 log_10_ CFU in bean (*P* < 0.001) and by 2.3 to 6.1 log_10_ CFU in radish (*P* < 0.001) (Fig. S7B). The increase in the bacterial population size during emergence was associated with a significant decrease (*P* < 0.001) in richness in bean (Fig. S7C and S8B). In contrast, no significant change in bacterial richness was detected in radish with either molecular marker (Fig. S5C and S9B).

10.1128/mbio.01648-22.9 FIG S8 Comparison of seed and seedling microbiota profiles. (A) The log_10_ CFU detected after seven days in our experimental system, which was composed of tubes filled with cotton and sterile water. (B) Average richness (estimated with *gyrB* ASVs) in the seeds and seedlings. (C) Taxonomic profile (order level, *gyrB*) of the seed and seedling microbiota. *P*-values were derived from a Wilcoxon rank sum test. Download FIG S8, TIF file, 0.3 MB. Copyright © 2022 Chesneau et al. 2022 Chesneau et al. https://creativecommons.org/licenses/by/4.0/ This content is distributed under the terms of the Creative Commons Attribution 4.0 International license.

10.1128/mbio.01648-22.10 FIG S9 Seed to seedling transmission. (A) Number of *gyrB* ASVs specifically detected on seeds (light color), seedlings (dark color), or both habitats (black). (B) Rank-abundance curves of *gyrB* ASVs in individual seeds and seedlings. (C) Comparison of the *gyrB* ASV relative abundance in the seeds and seedlings. Each dot corresponds to one ASV, which is colored according to its taxonomic affiliation at the order level. Download FIG S9, TIF file, 0.3 MB. Copyright © 2022 Chesneau et al. 2022 Chesneau et al. https://creativecommons.org/licenses/by/4.0/ This content is distributed under the terms of the Creative Commons Attribution 4.0 International license.

**FIG 5:**
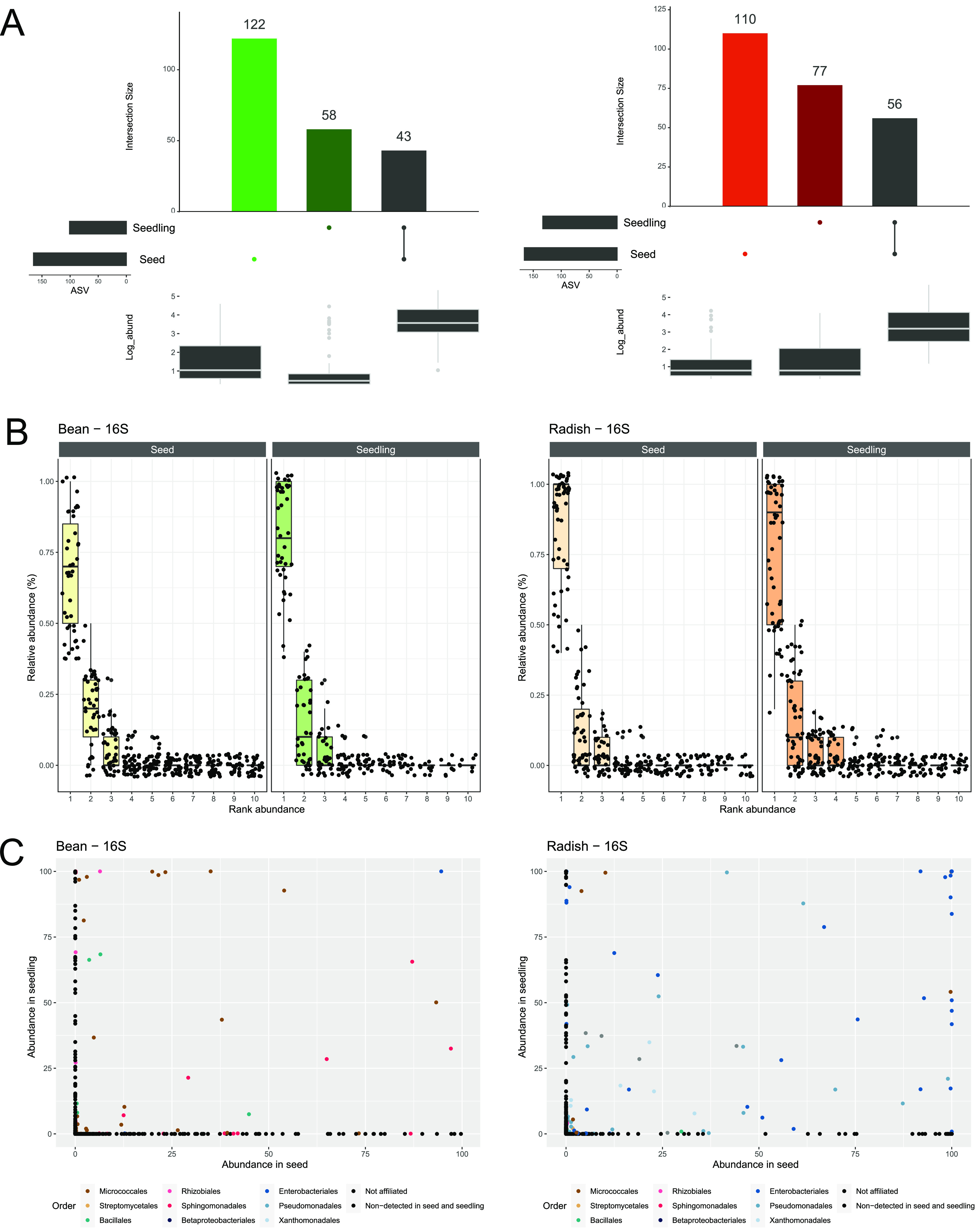
Seed to seedling transmission. (A) Number of 16S rRNA gene ASVs specifically detected on seeds (light color), seedlings (dark color), or both habitats (black). (B) Rank-abundance curves of 16S rRNA gene ASVs in individual seeds and seedlings. (C) Comparison of the relative abundance of 16S rRNA gene ASVs in seeds and seedlings. Each dot corresponds to one ASV, which is colored according to its taxonomic affiliation at the order level. Black dots represent ASVs that were not detected in seeds or seedlings.

About one fifth (43 out of 223) and one quarter (56 out of 243) of the bacterial ASVs associated with the bean and radish seeds were detected in seedlings using 16S rRNA genes (Fig. 5A). The use of *gyrB* reduced these proportions to 11% and 5.6% for bean and radish, respectively (Fig. S9A). More surprisingly, approximately 30% of the seedling-associated ASVs were not detected in seeds. The absence of CFU detection in our sterile experimental system (Fig. S8A) strongly suggested that these seedling-associated ASVs originated from seeds.

**(I) The initial abundance of seed-associated taxa does not explain its transmission to seedlings.** Next, we investigated whether the relative abundance of seed-borne ASVs was a predictor of seedling transmission. As had already been observed in experiment 2, each individual seed of both plant species was associated with one dominant ASV. According to the rank-abundance curves, one dominant ASV was also associated with individual bean and radish seedlings (Fig. 5B; Fig S9B). However, the dominant seed-associated ASV did not frequently become dominant in the corresponding seedlings (Fig. 5C; Fig S9C). Indeed, only 11 (out of 57) and 6 (out of 52) of the dominant seed-associated ASVs were dominant on radish seedlings, according to the 16S rRNA genes and *gyrB*, respectively. This value is even lower for bean, with 4 16S rRNA gene ASVs (out of 43) and 1 *gyrB* ASV that were dominant in both habitats. Overall, these results suggest that the initial abundance of seed-borne taxa is not predictive of its transmission to seedlings, especially in bean.

**FIG 6:**
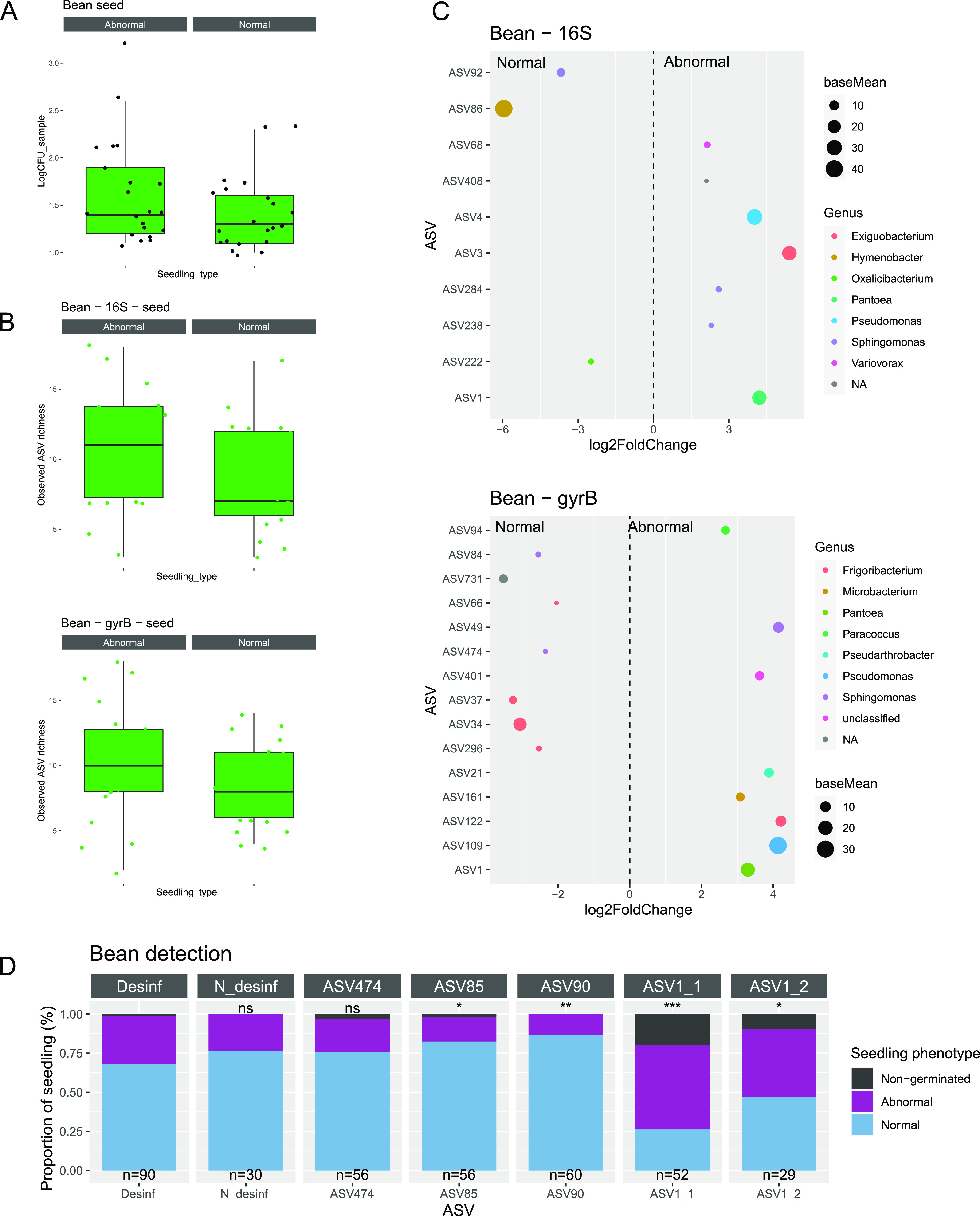
Impact of seed-borne ASVs on bean seedling phenotype. (A) Correspondence between the initial bacterial population size (log_10_ CFU) and the seedling phenotype. (B) Correspondence between the observed richness on seeds and the seedling phenotype. (C) ASVs with significant changes in relative abundance (*P* < 0.01) in normal and abnormal seedlings. Colors correspond to the taxonomic affiliation at the genus level. (D) Proportion of nongerminated seeds, normal seedlings, or abnormal seedlings after the seed inoculation of bacterial strains. All seeds were primarily disinfected before inoculation. Strains were representative of ASVs significantly associated with normal (ASV85 and ASV90) and abnormal seedlings (ASV1) or associated with both phenotypes (ASV474). Statistical tests were performed to compare the proportions of normal and abnormal phenotypes in disinfected seeds against each condition via the Chi-square test (ns = nonsignificant; *, *P* < 0.05; **, *P* < 0.01; ***, *P* < 0.001).

**(II) The identity of the seed-borne taxa affected seedling fitness.** Since seeds are colonized by a dominant taxon of variable identity, we investigated whether the identities of these dominant ASVs could impact seedling emergence. Seedlings were classified as normal or abnormal according to their morphologies. By using this classification scheme, 22 normal and 22 abnormal bean seedlings were obtained in addition to 40 normal and 10 abnormal radish seedlings (Fig. S7A). The initial bacterial population sizes and richness values observed from seeds were not good predictors of seedling phenotypes (Fig. 6A and B).

Next, changes in the relative abundance (RA) of seed-borne ASVs were investigated in the bean seeds but not in the radish seeds, as few seeds produced abnormal seedlings, and this did not allow for a balanced distribution. Overall, the RA of 6 16S rRNA gene ASVs and 8 *gyrB* ASVs were significantly enriched (*P* < 0.01, log2 FC > 2) in seeds that produced abnormal seedlings (Fig. 6C). The taxonomic affiliations of 4 of these 16S rRNA genes and *gyrB* ASVs were in agreement at the genus-level (Fig. 6C). On the other hand, the RA of 3 16S rRNA genes and 7 *gyrB* ASVs were significantly enriched in seeds that produced normal seedlings (Fig. 6C).

To validate this phenotype experimentally, we selected representative bacterial strains from our in-house bacterial collection on the basis of their *gyrB* sequences, and we inoculated these strains on bean seeds (Fig. 6D). We selected strains representative of ASVs (i) significantly enriched in seeds that produced abnormal seedlings (ASV1: Pantoea agglomerans), (ii) significantly enriched in seeds that produced normal seedlings (ASV85: Paenibacillus amylolyticus and ASV90: Bacillus aryabhattai), and (iii) not significantly enriched (ASV474: *Sphingomonas*). First, we validated that the employed seed surface-disinfection procedure did not impact seedling phenotype (Fig. 6D). As a control, a seed inoculation of ASV474 did not induce any difference in seedling phenotype. In contrast, seed-inoculation with two representative bacterial strains of ASV1 resulted in a significant (*P* < 0.05) increase of abnormal seedlings in comparison to surface sterilized seeds, mainly associated with rot symptoms. Inoculation of ASV85 and ASV90 increased the number of normal seedlings (Fig. 6D). Hence, we confirmed the seedling phenotype expected based on the RA of the ASVs.

## DISCUSSION

The primary objective of this study was to estimate the bacterial diversity of individual seeds during their development. Using a culture-enrichment procedure to perform community profiling at the individual seed level, we observed a low bacterial richness with medians of 7 and 4 bacterial ASVs being detected per bean and radish seed, respectively. A dominant bacterial taxon (>75% relative abundance) of variable identity (*n* = 224 ASVs) was associated with each individual seed. These results confirmed that seed colonization of bacteria is bottlenecked and that only one dominant taxon is able to colonize the seed, which is in agreement with the primary symbiont hypothesis (22).

### Whether the first microorganisms that colonize the seed during its development are the ones that will be established on mature seeds.

Overall, changes in community composition following the colonization of a nearly sterile habitat (e.g., seeds) by microorganisms is defined as primary succession (35). Primary succession is influenced by two main processes: (i) a change in limiting resources through time and (ii) the quantity of microorganisms that can colonize the habitat. In the case of the seed habitat, the first of these processes is associated with a change in the seed metabolites level, which occurs during its development (36), in addition to the impact of the microorganisms themselves via their metabolic activity and via their interactions with the host, which can impact primary succession (37). The second process (i.e., the quantity of microorganisms) is intimately linked to the seed transmission routes employed by the microorganisms, which act as sources of inocula for the seeds (7, 38). Although the destructive sampling procedure employed in this work does not allow for the direct monitoring of the dynamics of the assembly of the microbiota during seed development, indirect evidence suggested a contrasting response, depending on the plant species. For radish, bacterial phylogenetic diversity and taxonomic composition were stable over time, suggesting that pioneer taxa were maintained during seed maturation. In contrast, an increase in phylogenetic diversity together with a shift in bacterial taxonomic composition during bean seed development indicated the replacement of pioneer taxa during seed filling and maturation. Differences in succession profiles between plant species could be explained by differences in the availability of resources (i.e., radish seeds are oleaginous, whereas bean seeds are proteinous). This is an important process as demonstrated in soil, as soil with a high nutrient supply was subjected to a stronger priority effect than was soil with a low nutrient supply (39). However, this hypothesis should be tested with several genotypes per plant species.

### Main seed transmission routes.

Early seed colonizers employed two main routes: an internal seed transmission pathway via vascular tissues and a floral pathway that corresponds to the colonization of reproductive tissues via airborne inocula (40) or pollinators (17). It is difficult to specify the relative importance of these two pathways in the initial colonization of bean and radish seeds by bacterial taxa. Indeed, less than 20% of the seed-borne taxa were detected in the atmosphere, flowers, and stems, which probably reflected an undersampling of these source habitats in comparison to the seeds. A recent study has shown that approximately 10% and 40% of seed-borne taxa were derived from the floral pathway in bean and radish, respectively (7). In our study, the weak co-occurrence of bacterial ASVs in bean flowers and seeds confirmed that the floral pathway is of limited importance in beans. This difference in seed transmission pathways observed between plant species can be due to differences in pollination modes, with radish being allogamous and bean being autogamous (7). However, this hypothesis needs to be investigated experimentally. Regardless of the origin of early seed colonizers, significant variations in the identities of these taxa were observed between plant individuals. This interindividual variation has already been observed in radish seed microbial communities (18) and likely reflects the spatial heterogeneity of the distribution of microorganisms within the plot (41). Note that the entire study was performed after collecting the seeds aseptically from the fruits. In an agricultural ecosystem, mature seeds are collected mechanically and are mixed with plant residues. This passive dispersal of microorganisms could then impact the epiphyte composition of the seed microbiota (42).

### Ecological processes involved in the assembly of the microbiota at the individual seed level.

According to neutral models, the relative importance of the local replacement rate (selection or ecological drift) was higher than the immigration rate for both plant species during seed development. For bean, selection was the main driver of community assembly throughout the development of the seed. It is likely that the selection of pioneer taxa relies partly on host defense responses. Indeed, bypassing the activation of plant defense genes is key for the seed transmission of Xanthomonas citri pv. *fuscans* by the internal pathway (43). Moreover, the activation of the Medicago truncatula defense response was observed during seed filling, following the inoculation of X. campestris pv. *campestris* (44). In the later stages of seed development, we observed the replacement of pioneer taxa. This primary succession could be due to changes in the availability of water during seed maturation (32) or the in quantity and quality of organic carbon sources. In Fabaceae, seed filling is associated with an increase of the sucrose-to-hexose ratio (45), whereas seed maturation is characterized by an accumulation of Raffinose family oligosaccharides (RFO) at the expense of sucrose (46). Alternatively, other biotic interactions, notably the competition between microorganisms for resources and space, could also be involved in this selection. For instance, exclusionary interactions between seed-borne fungal isolates have been experimentally validated in Centaurea stoebe (47). For radish, the relative importance of the different ecological processes analyzed (selection, ecological drift, and dispersal) is less clear. Indeed, βNTI values were scattered on both sides of the threshold of −2 that is considered to be statistically significantly different from the null expectation (34). Therefore, it is likely that selection is less important in radish than in bean during seed development and that ecological drift is involved in the replacement rate observed. If this assumption is correct, this would be consistent with earlier results (18), in which drift was reported to be the key ecological process during the assembly of the bacterial communities of mature radish seeds.

### The initial abundance of seed-borne taxa as a predictor of their transmission to seedlings.

It is widely acknowledged that seed-borne taxa are not necessarily transmitted to seedlings (27). Seed borne bacterial population size is one parameter that could be involved in the efficiency of transmission to seedlings. For instance, a minimum population size of 10^3^ CFU per bean seed is required for an effective contamination of a seedling by *X. citri* pv. *fuscans* (48). According to our data, the bacterial population size was not a strong predictor of transmission to seedlings. This is in agreement with recent results obtained on rapeseed, which indicated that rare seed-borne or soilborne taxa were efficiently transmitted to seedlings (30). As the initial inoculum size does not guarantee successful transmission to seedlings, the difference in fitness between taxa (selection) appears to be a major driver of the assembly of the seedling microbiota. The competition for resources and space between bacterial taxa could be responsible for the selection of taxa in seedlings. Indeed, after the imbibition of the seed, a wide range and quantity of primary and secondary metabolites are exuded (49, 50). Labile substrates will be consumed first, which will favor copiotrophy taxa. These copiotrophic taxa are indeed reported as dominant in seedlings of bean and radish (29). Another interesting observation was that some of the dominant taxa in the seedlings were not found in the corresponding seeds. The community profiles of a significant portion of collected seeds (bean, 32%; radish, 42%) could not be estimated, as no CFU were detected on the culture medium employed, an observation already reported for the seeds of several plant species (20). It is unlikely that the seeds without detectable colonies could be considered sterile, as CFU were systematically obtained on each corresponding seedling, thereby justifying the use of seedlings for the detection of specific plant pathogens in the seed industry (a procedure called a grow-out test [21]). Alternatively, it is likely that some seed-associated bacterial populations were in a viable but nonculturable (VBNC) state, which corresponds to a low metabolic activity state without the formation of colonies on standard culture media. VBNC has been described in a number of seed-transmitted bacteria, including Clavibacter michiganensis pv. *michiganensis* (51) and Acidovorax citrulli (52). Why some populations enter the VBNC state in seeds and what the signals are that lift this state during germination-emergence are not yet known.

### The impact of dominant seed-borne taxa on seed vigor.

In our study, seed microbiota structure and bacterial biomass did not influence seed vigor. However, we identified changes in the RA of some specific ASVs in seeds that are correlated with abnormal seedlings. Moreover, we were able to validate the impact of seed-borne bacterial strains representative of these ASVs on seedling phenotype. Indeed, seed-inoculation of two bacterial strains affiliated with Paenibacillus amylolyticus and *Bacillus* resulted in an increase of normal seedlings. Firmicutes are well known for their beneficial impact on plant growth. For instance, seed-borne *Bacillus* species (23, 53) and *Paenibacillus* species (54) are known to significantly impact the seedling phenotype. In contrast, representative strains of Pantoea agglomerans caused an abnormal seedling phenotype, notably through root rot. *P. agglomerans* is a core seed microbiome taxon that is highly abundant and has been observed in almost all plant species studied to date (8). In bean, the occurrence of this taxon gradually decreased during seed filling and maturation. Therefore, symptoms caused by *P. agglomerans* in seedlings could occur when succession is not able to induce a low population level of this bacterial species during seed development.

### Conclusions.

This study provides key descriptive insights into the assembly of the seed microbiota, the identification of the origin of seed-associated taxa, the microbial transmission from the seed to the seedling, and the impact of seed borne taxa on seedling phenotype. These findings will inform the designs of seed microbiota engineering strategies by selecting (i) the most permissive seed transmission pathways and (ii) plant-beneficial bacteria that improve crop establishment. However, future studies will need to focus on the understanding of the mechanisms involved in seed microbial succession, especially at the community scale. These studies would provide essential information regarding the efficient manipulation of the seed microbiome to increase crop sustainability and productivity. We identified selection as the main driver of seed community assembly. However, to date, the natures of these selective processes remain to be explored. A promising future avenue would be to combine plant breeding efforts with seed microbiota engineering to maximize the benefits to plant heath.

## MATERIALS AND METHODS

### Experiment 1: Culture-based enrichment of seed-borne bacteria.

The low number of bacterial cells associated with each seed is a major limitation to the amplification and sequencing of molecular markers. To circumvent this limitation, a culture-based enrichment procedure was implemented.

This procedure was performed with seed samples (*n *= 8) of common bean (Phaseolus vulgaris var. Flavert) and radish (Raphanus sativus var. Flamboyant5). These samples corresponded to approximately 100 bean seeds (10 g) and 200 radish seeds (2 g). The seed samples were either ground or soaked. For grinding, seeds were crushed with a hammer until they were open. Per gram of tissue, 2 mL of phosphate-buffered saline (PBS, Sigma-Aldrich) supplemented with Tween 20 (0.05% vol/vol, Sigma-Aldrich) were added. The suspensions were homogenized in a lab blender (Stomacher, Mixwel, Alliance Bio Expertise) for 1 min. For soaking, the seed samples were soaked in 2 mL of PBS Tween 20 at 4°C under constant agitation (150 rpm) for 2.5 h and 16 h for the radish samples and the bean samples, respectively. These soaking procedures were developed by the International Seed Testing Association for the detection of bacterial pathogens located in seed internal tissues, such as Xanthomonas campestris pv. *campestris* on *Brassica* spp. (55) or Pseudomonas savastanoi pv. *phaseolicola* on bean (56). The grinding and soaking suspensions were centrifuged (4,000 × *g*, 10 min, room temperature [RT]). The supernatants were discarded, and the pellet was resuspended in 200 μL of PBS Tween 20. DNA extraction was performed with the NucleoSpin 96 Food Kit (Macherey-Nagel, Düren, Germany), following the supplier's recommendations.

In a second phase, 100 μL of soaking suspensions were serially diluted and plated on a panel of 9 media: bacterial screening medium 523 (BSM); casein peptone soybean flour peptone agar (CASO); nutrient broth agar (NB); Reasoner's 2A (R2A); r3 medium (R3M); tryptic soy agar, 1/10 strength (TSA10); tryptic soy agar (TSA100); tryptone yeast extract glucose (TYG) and yeast extract mannitol agar (YEM). All of the media were supplemented with cycloheximide (50 μg/mL, Sigma-Aldrich, Saint-Louis, MO, USA). The plates were incubated at 18°C for 5 days. The bacterial mats were scraped and collected by adding 2 mL of PBS Tween to the petri dishes, and they were stored at −20°C. DNA extraction was performed on the collected CFU suspension using the NucleoSpin 96 Food Kit.

Finally, the bacterial community profile was estimated before and after culture-enrichment on TSA10 (17 g/L tryptone, 3 g/L soybean peptone, 2.5 g/L glucose, 5 g/L NaCl, 5 g/L K_2_HPO_4_, and 15 g/L agar). A total of 12 seed samples per plant species were collected and analyzed. The 24 samples were soaked as previously described. Half of the suspension (100 μL) was stored at −20°C, while the other half (100 μL) was serially diluted and plated on TSA10. The plates were incubated at 18°C for 5 days. DNA was extracted using a NucleoSpin 96 Food Kit as previously reported.

### Experiment 2: Community assembly during seed development at the individual seed level.

A field trial was performed at the experimental station of the National Federation of Seed Multipliers (FNAMS, 47°28'012.42''N, −0°23'44.30''W, Brain-sur-l'Authion, France). Bean and radish were grown in separate blocks (5 m × 10 m) at a density of 8 bean plants and 5 radish plants per square meter.

Flowers from 5 plants at the blooming stage were collected, with all flowers from one plant corresponding to one independent sample. The flowers were soaked in 2 mL of PBS Tween per gram of tissue and then crushed in a lab blender (Stomacher, Mixwel, Alliance Bio Expertise, Guipry, France) for 2 min.

Individual seeds from 5 plants of bean and radish were collected at different stages of the seed development process (Fig. S1). The number of seeds harvested per plant was determined by the number of seeds available on the plant at the given stage of development. We were then able to sample a total of 96 and 144 seeds per radish and bean plant, respectively. Individual seeds were soaked in 750 μL and 1.5 mL of PBS Tween for 2.5 h and 16 h for radish and bean, respectively. The seed water content was estimated at each sampling stage on a bulk of 15 seeds by weighing the sample before and after drying (96°C for 3 days).

The vascular flow of the stem of each individual plant was collected as follows. A 2 cm section of stem from the bottom of the plant was cut, and the outer surface was disinfected using 70% ethanol. Then, the first layers of epidermis were removed using a sterile scalpel. The resulting piece of stem was incubated horizontally for 2.5 h in 2 mL of PBS Tween under constant agitation (200 rpm) at 4°C.

Finally, airborne bacteria were collected at each sampling stage. To achieve this, a passive sampling scheme was performed with TSA10 plates placed at a distance of 10 to 20 cm from the flowers. 6 TSA10 plates were positioned homogeneously within the plot for 3 h.

All of the plates were incubated at 18°C for 5 days. DNA was extracted using a NucleoSpin 96 Food Kit as previously reported.

### Experiment 3: Community dynamics during emergence at the individual level.

At the last sampling point of experiment 2, 32 mature seeds per plant (*n* = 5 plants) were collected (*n* = 160 seeds). The seeds were soaked in 750 μL and 1.5 mL of PBS Tween. After collecting the resulting suspension, the seeds were placed in sterile tubes filled with cotton that was moistened with 4 mL of sterile water. The tubes were incubated in a growth chamber (photoperiod, 16 h/8 h; temperature, 22 to 25°C) to obtain 4-day-old radish seedlings and 6-day-old bean seedlings. The seedlings were placed individually in a plastic bag, crushed, and resuspended with 2 mL of sterile water. Suspensions of seeds and seedlings were serially diluted and plated on TSA10. DNA was extracted as previously reported. The sterility of our experimental system was assessed by collecting cotton from seedless tubes (*n* = 30).

### Construction and sequencing of amplicon libraries.

A first PCR amplification was performed with the primer sets 515f/806r (57) and gyrB_aF64/gyrB_aR553, which target the V4 region of 16S rRNA genes and a portion of *gyrB*, respectively. Polymerase chain reactions (PCRs) were performed with a high-fidelity *Taq* DNA polymerase (AccuPrime *Taq* DNA Polymerase System, Invitrogen, Carlsbad, CA, USA) using 5 μL of 10× buffer, 1 μL of forward and reverse primers (gyrB_aF64/gyrB_aR553 [100 μM]; 515f/806r [10 μM]), 0.2 μL of *Taq*, and 5 μL of DNA. Cycling conditions for 515f/806r were composed of an initial denaturation step at 94°C for 3 min, followed by 35 cycles of amplification at 94°C (30 s), 50°C (45 s), and 68°C (90 s), and a final elongation at 68°C for 10 min. The cycling conditions for gyrB_aF64/gyrB_aR553 were identical, with the exception of the annealing temperature (55°C instead of 50°C). The amplicons were purified with magnetic beads (Sera-Mag, Merck, Kenilworth, New Jersey). A second PCR amplification was performed to incorporate Illumina adapters and barcodes. PCR cycling conditions were as follows: denaturation at 94°C (2 min), 12 cycles at 94°C (1 min), 55°C (1 min), 68°C (1 min), and a final elongation at 68°C for 10 min. The amplicons were purified with magnetic beads and pooled. Concentration of the pool was monitored via quantitative PCR (KAPA Library Quantification Kit, Roche, Basel, Switzerland). Amplicon libraries were mixed with 5% PhiX and sequenced with four MiSeq reagent kits v2 for 500 cycles (Illumina, San Diego, CA, USA). A blank extraction kit control, a PCR-negative-control, and a PCR-positive-control (Lactococcus piscium, a fish pathogen that is not plant-associated) were included in each PCR plate.

### Microbial community analysis.

All scripts and data sets employed in this work are available on GitHub (https://github.com/martialbriand/IRHS_EmerSys). Briefly, primer sequences were removed with cutadapt version 1.8 (58). Fastq files were processed with DADA2 version 1.6.0 (59). Chimeric sequences were removed with the removeBimeraDenovo function. The taxonomic affiliation of ASVs was performed with a naive Bayesian classifier (60) against the Silva 132 taxonomic training data or an in-house *gyrB* database, which is available upon request.

Diversity analyses were conducted with Phyloseq version 1.32.0 (61). Sequences derived from the 16S rRNA genes that were unclassified at the phylum-level (0.002%) or were affiliated with Chloroplasts (0.11%) or Mitochondria (0.05%) were removed. Since the primer set gyrB_aF64/gyrB_aR553 primers can sometimes coamplify *parE*, a paralog of *gyrB*, the *gyrB* taxonomic training data also contained *parE* sequences. ASVs affiliated with *parE* or unclassified at the phylum-level were removed. Sequences were aligned with DECIPHER version 2.16.1 (62), and neighbor joining phylogenetic trees were constructed using Phangorn version 2.5.5 (63). The identification of sequence contaminants was assessed using decontam version 1.8.0 (64).

Alpha diversity metrics (richness and Faith’s phylogenetic diversity) were measured after rarefaction at 4,000 reads per sample for all data sets and for both markers (Fig. S2). Changes in the bacterial phylogenetic composition were estimated with weighted UniFrac distances (65) on log_10_ +1 transformed values. A permutational multivariate analysis of variance was performed with the adonis2 function of vegan 2.5.6. Rank-abundance curves were computed using BiodiversityR 2.12.3. The number of ASVs shared between specific contrasts were visualized with UpSetR 1.4.0 (66). The annotations of the phylogenetic trees were performed with iTol 6.1 (67).

The relative importance of niche-or neutral-based processes in community assembly was estimated with abundance-based β-null deviation measures. Briefly, a pairwise comparison of seed communities, aggregated at the individual plant level, was performed using the β-mean-nearest taxon distance (βMNTD) (68). The βMNTD represents the phylogenetic distance between each taxa (here, each ASV) in one local community and its closest relative in a second community. The resulting βMNTD values were compared to the null distribution obtained via the randomization of the phylogenetic position of the ASVs. The deviations of the βMNTD values from the mean of the null distribution was estimated using the β-Nearest Taxon Index (βNTI, [33]). βNTI values of <−2 or >2 indicate that the observed phylogenetic turnover between a pair of communities is primarily driven by selection (33).

### Analysis of species-proportion distributions.

When considering the set of all ASVs observed on a plant, only a few of them are detected in each seed. This is probably caused by censoring induced by the measurement process, but it also probably comes from important spatial heterogeneity. Therefore, we proposed a dedicated approach to provide a species-proportion analysis in seeds at the plant level for the most abundant species (>0.1% of the total ASV abundance). We used the neutral model of Solé and coworkers (69) at the seed level, where all ASVs were assumed to be totally equivalent and driven by immigration (probability 0 < μ < 1) and internal random replacement (probability 0 < C < 1). The abundances in each seed follow a beta-binomial (BB) distribution with parameters $N_{c}$ (the local ASV population size in the seed), $\alpha_{c},\beta_{c}$, where S is the typical species pool size available for immigration in the vicinity of each seed, and I is the ratio of the per capita immigration rate over the per capita replacement rate, $I_{c}=\frac{\mu }{\left(1-\mu \right)C^{}}\left(N_{c}-1\right)$. To obtain the distribution of seed-borne proportions at the plant level, we assumed that N followed a uniform distribution whose range [Nmin, Nmax] was determined as the 0.1 to 0.9 quantiles of the distribution of the total ASV number in the seeds at a given date. The other parameters ($S,R=\frac{\mu }{\left(1-\mu \right)C^{}}$) were assumed to be plant dependent (see supplementary material - Text S1). We computed from the data the proportions of ASVs in each seed at a given date on a given plant, evaluated the first three empirical moments of the mean at the plant level, and performed a generalized moment estimation to obtain an estimated value for S, R, and the expected I, as well as for the value of the error criterion (see Supplemental Methods for more details). All computations were coded in R, and the Wilcoxon test (resp. pairwise Wilcoxon rank test with the Benjamini-Hochberg correction) was used for simple (resp. multiple) comparisons.

10.1128/mbio.01648-22.1 TEXT S1 Supplemental methods. Download Text S1, DOCX file, 0.02 MB. Copyright © 2022 Chesneau et al. 2022 Chesneau et al. https://creativecommons.org/licenses/by/4.0/ This content is distributed under the terms of the Creative Commons Attribution 4.0 International license.

### Seedling phenotype.

Seedling phenotype was estimated via rules established by the International Seed Testing Association (https://www.seedtest.org/en/home.html). Briefly, a seedling was considered abnormal if at least 50% of the cotyledons or leaves were necrotic or rotten, if the hypocotyl or epicotyl were deformed, or if the root system was absent, stunted, or rotten.

Changes in the relative abundance of ASVs were assessed with DESeq2 v 1.28 (70). Representative bacterial strains of ASVs significantly enriched in abnormal or normal seedlings were selected from an in-house bacterial culture collection that was derived from bean seeds.

The bean seeds were surface-sterilized after 1 min of sonication (40 Hz), soaked for 1 min in 96° ethanol, 5 min in 2.6% sodium hypochlorite, and 30 sec in 96° ethanol, and then rinsed 3 times with sterile water. The seeds were dried on paper and placed in a bacterial inoculum from a fresh 24-h culture that was calibrated at DO_600nm_ = 0.01 (approximately 10^7^ CFU/mL). The seeds were then dried on paper. In order to check the inoculation of the seeds, half of the dried seeds were individually macerated in a prefilled plate of PBS Tween. The other half of the seeds were placed in sterile tubes with cotton and moistened with 4 mL of water at the level of one seed per tube. The phenotypes of the seedling were evaluated after 6 days in a growth chamber (photoperiod, 16 h/8 h, temperature, 22 to 25°C). We also analyzed the seedling phenotypes of nondisinfected seeds to see the impact of disinfection on the seedling phenotypes.

To confirm the transmission of the inoculated strains, for each phenotype, we gathered seedlings in three plastic bags, one for nongerminated seeds, one for normal seedlings, and the last one for abnormal phenotypes. The batches were crushed and resuspended with 2 mL of sterile water per individual. The suspensions of seedlings were serially diluted and plated on TSA 10%. After 5 days, we performed *gyrB* PCR amplification of the representative members of each plate with the gyrB_aF64/gyrB_aR553 primers. The PCR products were sent to GenoScreen for Sanger sequencing.

### Data availability.

The data sets supporting the conclusions of this article are available in the European Nucleotide Archive under the accession number PRJEB45079. All R scripts employed in this work are available on GitHub (https://github.com/martialbriand/IRHS_EmerSys).
