# Supplementary material for: Single Seed Microbiota: Assembly and Transmission from Parent Plant to Seedling
Source: mBio. 2022 Oct 12;13(6):e01648-22. doi: 10.1128/mbio.01648-22 (PMC9765463; doi:10.1128/mbio.01648-22)
Supplement: TEXT S1 [file mbio.01648-22-s0010.docx]

**Supplemental methods : Species-proportion distribution analysis.**

We performed a qualitative analysis of the species-proportion distributions for both plant species. We focused on species thought to be actually representative of within seed microbiota, so microbial species whose abundance was less than 0.1% of the total abundance detected in one seed were discarded.

For each day and each plant, we computed the *empirical moments* of the observed ASV proportions. When considering the set of all ASV observed on a plant, only a few of them are detected in each seed. This is probably caused by censoring induced by the measurement process, but it probably also comes from spatial heterogeneity. This is why we computed the ASV proportions (*f*) for each seed related to the ASVs detected on that seed, and discarded the null frequencies for unobserved ASVs. Defining $I_{plant}$and $J_{seed}$ as the set of seeds observed at a given date on a given plant and the set of ASV *observed at least one time* in this seed on that plant at the same date respectively, we obtain for each plant the following expressions for the first three empirical moments

$${\overline{m_{1,2,3}}}_{plant}=\sum_{i\in I_{plant}} \frac{1}{\left| I_{plant} \right|}\sum_{j\in J_{i}} \frac{{f_{ij}}^{1,2,3}}{\left| J_{i} \right|}$$

We compared these moments with those obtained with a neutral distribution derived from (69). These authors proposed the use of neutral distribution as a crude first order approximation of the species-abundance distributions in complex ecosystems. This distribution is the stationary distribution of a neutral model, involving local species communities (for us, a microbial community associated to a seed in a plant) and a “local” regional pool (for us, a plant associated community in the vicinity of a seed). Here, assumption of neutrality is defined as follows (see [Laroche 2020]): (i) local per capita rates of birth and death are identical for all individuals, whatever their species, (ii) immigration in the local community occurs through random sampling of individuals from the regional pool, (iii) all these rates depend on the local community size only. Moreover, it is assumed in this model that the local community is saturated (constant size *N*>0), and the typical local species pool size (the number of ASV available for immigration from the plant and its close environment in the vicinity of a seed) is *S*>0.

Two phenomena are accounted for, immigration (probability $\mu$, such that $0<\mu<1$) and local competition, modelled through a parameter $C^{*}$, representing the percentage of ASV with which an ASV competes, with equal probability to win. This parameter is also called the connectivity of the local community. The corresponding probability to win (replacement probability) is $C=1-\left( 1-C^{*} \right)^{2}$.

Standard results in ecological theory (see [Laroche 2020]) lead to the definition of the ratio of the per capita immigration rate ($\mu$) over the per capita replacement rate ($\left( 1-\mu\right)C/\left( N_{c}-1 \right)$) in a community (seed) c of size $N_{c}$.

$$I_{c}=\frac{\mu}{\left( 1-\mu\right)C}\left( N_{c}-1 \right)$$

This is the relevant ecological parameter in these models, measuring the relative strength of immigration and internal competition or selection dynamics that shape the species abundance in the community, together with the local species pool size S. The species abundance distribution $P$ is then given by a Beta-binomial distribution with parameters $N_{c},\alpha_{c},\beta_{c}$ expressed as

$$\alpha_{c}=\frac{I_{c}}{S},\beta_{c}=I_{c}\frac{\left( S-1 \right)}{S},PBb\left( n \right)=\left( \begin{matrix} N_{c} \\ n \end{matrix} \right)\frac{\Gamma\left( n+\alpha_{c} \right)\Gamma\left( N_{c}-n+\beta_{c} \right)}{\Gamma\left( N_{c}+\alpha_{c}+\beta_{c} \right)}\frac{\Gamma\left( \alpha_{c}+\beta_{c} \right)}{\Gamma\left( \alpha_{c} \right)\Gamma\left( \beta_{c} \right)}$$

**We modified this model to compute the species-abundance distribution at the plant level for a given day.** To this end, we hypothesized that for seeds in which at least one microbe was detected, the distribution of total number of detected ASVs in all seeds was an acceptable proxy of the distribution of $N_{c}$ accounting for natural variability and variability induced by the sampling process, but ignoring cultivation bias. Moreover, after inspection of the data, we assumed that for each observation date d

$N_{c}\sim uniform\left( N_{mind},N_{maxd} \right)$

where $N_{mind},N_{maxd}$ depends on the sampling date but are the same for all plants.

Therefore, the marginal species abundance distribution at date d for seeds of plant p is given by

$\overline{P}_{N_{mind},N_{maxd},S,\frac{\mu}{\left( 1-\mu\right)C}}\left( n \right)=\sum_{N>0,N\geq n} PBb\left( n\vee N,S,\frac{\mu}{\left( 1-\mu\right)C} \right)Punif\left( N | N_{mind},N_{maxd} \right)$where $PBb$ and $Punif$ are the Beta-binomial and uniform (on integer) probability functions.

We may notice that the model actually depends on the ratio

$$R=\frac{\mu}{\left( 1-\mu\right)C}$$

and the parameter $S$ and $N_{mind},N_{maxd}$. As we actually only observe proportions of ASVs present within each seed, we compute the three first moments of positive proportion distribution at the plant level for this model:

$$m_{1}=E\left( f\vee1\geq f>0 \right)=\sum_{n>0} f\sum_{N>0,\frac{n}{N}=f} \frac{PBb\left( n | N,S,R \right)}{\left( 1-PBb\left( 0 | N,S,R \right) \right)}Punif\left( N | N_{mind},N_{maxd} \right)=\sum_{N>0} \left( \sum_{N\geq n>0} \frac{n}{N}\frac{PBb\left( n | N,S,R \right)}{\left( 1-PBb\left( 0 | N,S,R \right) \right)} \right)Punif\left( N | N_{mind},N_{maxd} \right)=\sum_{N>0} \frac{1/S}{\left( 1-PBb\left( 0 | N,S,R \right) \right)}Punif\left( N | N_{mind},N_{maxd} \right)$$

$$m_{2,3}=E\left( f^{2,3}\vee f>0 \right)=\sum_{N>0} \left( \sum_{N\geq n>0} \left( \frac{n}{N} \right)^{2,3}\frac{PBb\left( n | N,S,R \right)}{\left( 1-PBb\left( 0 | N,S,R \right) \right)} \right)Punif\left( N | N_{mind},N_{maxd} \right)$$

Based on this model, for each date we used the total number of ASVs to choose $N_{mind},N_{maxd}$ separately. After plotting the quantiles for each date, we observed that we could reasonably assume a uniform distribution on the interval corresponding to the 0.1 and 0.9 quantiles. Then, for a given set of parameters we simulated 1000 values of $N$ and computed the moments according to the above formula.

We performed a **generalized moment estimation** of the parameters ($S,R$) to fit the observed moments for each plant. For this we screened the following parameter grid (whose range was selected by preliminary trial):

$$S=\left\{ 2,3,\ldots,99,100 \right\},$$

$$R=\left\{ 0.000001,0.000002,0.000003,\ldots,0.000248,0.000249,0.00025 \right\}$$

and selected for each plant the set of parameters that minimize the quantity

$$\sqrt{\left( m_{1obs}-m_{1} \right)^{2}+\left( m_{2obs}-m_{2} \right)^{2}+\left( m_{3obs}-m_{3} \right)^{2}}$$

We also tested a weighted version of this criterion, under the form

$$\sqrt{\left( \frac{m_{1obs}-m_{1}}{m_{1obs}} \right)^{2}+\left( \frac{m_{2obs}-m_{2}}{m_{2obs}} \right)^{2}+\left( \frac{m_{3obs}-m_{3}}{m_{3obs}} \right)^{2}}$$

The optimal parameters were then used to compute the ecologically relevant ratio

$$I_{plant}=R\left( E\left( N \right)-1 \right)=\frac{\mu}{\left( 1-\mu\right)C}\left( E\left( N \right)-1 \right)$$

where $E(N)$ is the expected value of N under the uniform distribution.

**References:**

[Laroche 2020] Laroche, Fabien, et al. "Analyzing snapshot diversity patterns with the Neutral Theory can show functional groups’ effects on community assembly." Ecology 101.4 (2020): e02977.
